# Effect of Local Ketamine Subcutaneous Injection at the Incision Site in Reducing the Postoperative Pain Score after Transabdominal Hysterectomy

**DOI:** 10.1155/2023/7782847

**Published:** 2023-11-04

**Authors:** Negar Eftekhar, Babak Eslami, Amir Hossein Orandi, Leila Chabouk, Fahimeh Ghotbizadeh Vahdani, Hoda Mohammad khani, Laya Amoozadeh

**Affiliations:** ^1^Department of Anesthesiology and Intensive Care, Imam Khomeini Hospital Complex, Tehran University of Medical Sciences, Tehran, Iran; ^2^Imam Khomeini Medical Complex, Tehran University of Medical Sciences, Tehran, Iran; ^3^Obstetrics and Gynecology Department, Imam Khomeini Medical Complex, Tehran University of Medical Sciences, Tehran, Iran

## Abstract

**Background:**

Pain control after operations is essential in decreasing the patient recovery period and potential morbidity. Prescribing opiates is very effective, but significant side effects accompany them. This study aims to examine the effect of local ketamine infiltration in decreasing pain intensity in patients undergoing transabdominal hysterectomy.

**Methods:**

In this double-blind, randomized, controlled clinical trial, a total of 92 patients undergoing transabdominal hysterectomy aged 30–60 years were selected and divided into two intervention and control groups randomly. For the intervention group, ketamine was injected subcutaneously into the incision site at a dose of 0.5 mg/kg after the operation. In the control group, 5 mg normal saline was used in the same method. Postoperative pain intensity was measured using the visual analog scale (VAS: 0–10). The pain score and dose of administered opioids were documented at 1, 2, 4, 6, 12, and 24 hours and compared between the two groups.

**Results:**

Postoperative pain intensity was significantly lower in the intervention group than in the control group, except for hour 24. The mean amounts of administered opioids were significantly lower in the intervention group at hours 6 and 12, as well as the total amount of used opioids, and no significant side effects were documented.

**Conclusion:**

Local ketamine subcutaneous injection in the incisional site is effective and is a safe procedure for reducing pain scores in patients who underwent a transabdominal hysterectomy.

## 1. Introduction

The incidence of postoperative pain (PP) after abdominal surgery has been reported between 0% and 34% [1, 2]. Postoperative pain is an unpleasant physical and mental experience, especially for a patient undergoing surgery. It may increase anxiety, extend the normal healing process, and bring many other unpleasant consequences [3]. The main component of PP is neuropathic, which could intervene not only in the intensity of the perception of immediate postsurgical pain but also could prolong the healing of the wound [4, 5]. Despite this, adequate PP control could result in accelerated healing and ward off further complications [6].

Overusing narcotic analgesics in such patients could bring a lot of significant side effects, a condition that has persuaded scientists to use safer alternative pain-control methods and medications [7–9]. Also, the doses of opioids and nonsteroidal anti-inflammatory drugs for intravenous (IV) analgesia are limited in patients with hepatic disease. Low-dose ketamine is frequently used [10, 11].

Ketamine, an N-methyl-D-aspartate (NMDA) receptor antagonist, is a potentially effective and safe medication associated widely pre- and postoperatively to decrease pain intensity and has been reported to be associated with a significant decrease in narcotics use for this purpose [12]. There is clear evidence that ketamine reduces the intensity of acute postsurgical pain [10, 13, 14]. Also, NMDA has a key role in central sensitization causing PP, which is manifested as secondary hyperalgesia. Ketamine is one of the few clinically available NMDA receptor antagonists and is widely used positively for postsurgical analgesia [10, 14]. Acting through inhibiting NMDA receptors located on the postsynaptic membrane of the posterior spinal cord, ketamine prevents the transfer of pain signals via dedicated pathways from peripheral tissues towards the central nervous system (CNS) [15]. It has an analgesic role by inducing anti-inflammatory effects in the CNS [16]. Besides its analgesic effects, ketamine is considered an essential medication in patients undergoing surgeries because, unlike many other conventionally employed analgesics in these patients, ketamine does not affect the respiratory system and brings further amnestic and relaxing benefits [17]. After a cesarean section, hysterectomy is the second most common gynecological surgery in the United States (US) [18] since almost 40% of females undergo hysterectomy before the age of 60 years due to various underlying causes such as uterine cancer and fibroma. Like in the US, hysterectomy is also one of Iran's most commonly performed gynecological operations [19]. Using ketamine as an effective analgesic after hysterectomy has been studied previously [20, 21], but the available reports are heterogeneous, and further studies are needed. This study aims to examine the effect of subcutaneous ketamine infiltration on pain control within the first 24 h after abdominal hysterectomy.

## 2. Methods and Materials

### 2.1. Patients

This double-blind, placebo-controlled, clinical trial was conducted to examine the analgesic effect of ketamine infiltrated locally in the incisional site in transabdominal hysterectomy. After the approval of the study protocol by the Ethics Committee of the Tehran University of Medical Sciences and obtaining written consent from the participants, a total of 100 patients undergoing abdominal hysterectomy (American Society of Anesthesiologists, ASA classes I and II) referred to Valiasr Teaching Center from 2014 to 2015 were selected to participate in this study. The age range of patients was considered between 30 and 60 years.

Exclusion criteria were patient refusal, patients with cardiac problems, schizophrenia, seizure, hypertension, history of drug/alcohol addiction, brain space-occupying lesion, and intracerebral pressure elevation.

To detect one level decrease in the pain score according to the visual analog scale (VAS) and to assume *α* = 0, *β* = 0.01, and power = 80%, the sample size was estimated at 46 patients in each group, which was augmented to 50 patients to consider possible losses.

### 2.2. Methods

Patients were classified into two case and control groups according to the type of analgesia they received. First, each patient received a random computer-generated code (available at https://Randomization.org). Accordingly, patients were randomized into either case or control groups. Induction and maintenance of anesthesia were performed similarly in both groups, including intravenous midazolam (0.01 mg/kg) and fentanyl (3 mic/kg) as premedication agents and intravenous sodium thiopental (5 mg/kg) followed by atracurium (0.01 mg/kg) as induction agents. Then, the patients underwent laryngoscopic tracheal intubation and mechanical ventilation after that. Anesthesia was continued using continuous infusion of propofol (100–150 mic/kg), fentanyl, and atracurium when needed.

At the end of the operation and just before wound closure, ketamine (Rotexmedica, Germany) was infiltrated subcutaneously (0.5 mg/kg in 5 ccs) in the incisional site of patients in the case group. Instead of ketamine, normal saline (Abidi, Iran) was used similarly in the control group. Patients were then transferred to the recovery after extubation and antagonizing relaxing effects of neuromuscular blockers. Pain intensity was recorded at hours 1, 2, 4, 6, 12, and 24 after surgery in patients who were instructed about how to refer to pain using the visual analog scale (VAS), which allows identification and qualification of neuropathic symptoms reflecting spontaneous or paroxysmal pain and evoked pain, which was considered positive with scores higher than zero. Accordingly, 0 indicated no pain and 10 showed the most severe pain experienced by the patient [22]. In patients with severe PP intensity (i.e., VAS > 5), using additional narcotics (morphine as rescue analgesia) was allowed. Besides PP intensity, the dose of narcotics requested by patients and administered as single doses or via PCA pump within the first 24 h postoperation was also recorded.

### 2.3. Statistics Analysis

SPSS software (IBM Corp. Released 2011. IBM SPSS Statistics for Windows, Version 20.0. Armonk, NY: IBM Corp.) was used for statistical analysis. The quantitative variables were expressed as the mean ± standard deviation, and the qualitative variables were defined as frequency (%). Repeated measures analysis (RMA) and the independent samples *t*-test were used for comparisons. A normal distribution of the numerical data was assured using the Kolmogorov–Smirnov test. A *P* value <0.05 was considered statistically significant.

## 3. Results

All the initially recruited 92 patients completed the study protocol. The mean age of patients was 49.41 ± 7.10 years in the case group and 49.25 ± 6.98 years in the control group (*P*=0.80). The two groups were also comparable in the mean weight (67.41 ± 7.24 kg in cases and 65.52 ± 7.21 kg in controls, *P*=0.20).

Pain intensity at different intervals in cases and controls is summarized and compared between the two groups in Table 1. Accordingly, there were significant differences between the two groups at all-time intervals except for that on 24 h.

Among different time intervals, the least pain intensity in the case group was documented on 24 h, followed by those recorded on 4 and 6 hrs. In the control group, the least pain intensity was observed on 24 h and then at 4 h. The most severe pain was on 12 h in the case group and at 1 h in the control group, indicating that the analgesic effect of ketamine was more evident during the first postoperative hours. Still, despite documenting the least pain intensity on 24 h in both the case and control groups, there was no significant difference between them in this regard. In other words, ketamine exerted a more prominent analgesic effect during the early postoperative hours.

The mean doses of administered narcotics at different intervals in cases and controls are summarized and compared between the two groups in Table 2.

Accordingly, the mean dose of administered morphine was lower in cases than in controls at all intervals. However, statistically, significant differences were present only on 6 and 12 hrs. Overall, ketamine use decreased narcotic requirements significantly.

Although the number of narcotics requested by patients decreased by age, this correlation was not statistically significant (*r* = −0.04, *P*=0.67). So, it could be concluded that younger patients requested narcotics more frequently than their older counterparts.

## 4. Discussion

According to the results of the present study, during the first 24 h after abdominal hysterectomy, the subcutaneous administration of ketamine in the incisional site postoperative pain intensity has decreased significantly compared to those who received a placebo. This pain reduction was significant on hours 1, 2, 4, 6, and 12 after surgery, but the difference did not reach a considerable level despite less severe pain on 24 h in cases. In addition, although the morphine administered in cases was lower than that in controls at all-time intervals, there was no significant difference between the two groups on hours 1, 2, 4, and 24 postoperation. Also, we did not encounter any significant complications in this study.

Acute pain after the operation is a complex physiologic reaction due to tissue injury, visceral extension, or the underlying disease itself. This pain may lead to complications such as compromised respiratory ventilation and resultant atelectasis. In addition, pain could restrict patients' movements and increase the risk of deep vein thrombosis due to immobility [23].

A retrospective control-case study performed on 101 patients who underwent different types of abdominal surgeries compared the combination of epidural technique and general anesthesia with general anesthesia alone and found a PP incidence [1, 24]. In 85 patients with rectal adenocarcinoma surgery performed with different analgesia regimes, including ketamine, a prospective study with control of VAS reported a PP incidence between 0% and 48%.

In a single-blind study by Karaman et al. [25] that was carried out to examine the pain-reducing effect of ketamine in three 20-patient groups, intravenous bolus ketamine (0.4 mg/kg) was administered immediately after hysterectomy in the two intervention groups. Intravenous morphine was also administered after the operation if required. Pain intensity was documented on hours 1, 2, 3, 4, 8, 12, and 24 after surgery. According to their findings, there were no significant differences between the three groups regarding pain reduction within the first 24 h and the total dose of morphine used postoperatively [25]. The authors recommended further studies using higher doses of ketamine and other types of surgeries.

In a double-blind study, Dahl et al. [26] examined the pain-reducing effect of ketamine after abdominal hysterectomy. For this purpose, 89 patients were divided randomly into three equal groups who underwent similar protocols of general anesthesia. In the first group, normal saline (0.04 mg/kg) was administered intravenously before incision and after wound closure. In the second group, ketamine (0.4 mg/kg) was administered intravenously before incision, followed by intravenous normal saline after wound closure and vice versa in the third group. The results showed that the pain severity during the first hour after surgery was significantly lower in the third than in the other two groups. They finally concluded that a single dose of ketamine produces a short-term postoperative analgesic effect, with no significant difference between the groups after the first hour of postoperation [26].

Behaeen et al. [27] examined the pain-reducing effect of subcutaneous ketamine after cesarean section. In this series, 60 candidates for elective cesarean section aged 18–26 years were randomized into three 20-patient groups. In the first group, ketamine (0.5 mg/kg) was used subcutaneously before incision, and normal saline was administrated in the same fashion after wound closure. In the second group, normal saline and ketamine (0.5 mg/kg) were administered subcutaneously before incision and after wound closure, respectively. In the third group, only normal saline was administered before incision and after wound closure. The time for the first postoperative analgesic demand was longer, and the mean dose of administered analgesics during the first 24 h was lower in groups 1 and 2 than in the third group. Pain intensity on hours 2, 4, 6, and 12 postoperation was significantly lower in the first two groups than in the third group. No significant difference was found between the first two groups in terms of the mentioned variables.

In another double-blind, randomized study by Joel et al. [28], the pain-reducing effect of intravenous ketamine was evaluated in delivery. A total of 70 pregnant women who were candidates for vaginal delivery were randomized into two groups. Initially, both groups received intramuscular meperidine for pain reduction. Then, in the first group, intravenous ketamine (0.2 mg/kg) was infused within 30 minutes, followed by the same dose infused continuously until delivery. The same dose of normal saline was used in the second group. Pain reduced significantly in ketamine receivers, and in over 60% of the women, the pain was relieved entirely an hour after delivery. At the same time, no significant changes were documented in either mothers' hemodynamic status or fetal heartbeat.

In the double-blind study of Mohamed et al. [29], ninety patients underwent abdominal hysterectomy. There were three groups with local wound infiltration with bupivacaine plus ketamine or dexmedetomidine. They showed that local wound infiltration with ketamine or dexmedetomidine added to bupivacaine had an opioid-sparing effect, delayed first request of rescue analgesia, and attenuated postoperative stress response, especially with ketamine in these patients.

## 5. Conclusion

Although it was first used purely as an anesthetic, ketamine is making a particular resurgence in managing postoperative pain. As mentioned above, many studies have demonstrated significant effectiveness in controlling postoperative pain, increasing the time to first analgesic request, decreasing overall opioids required, and demonstrating fewer opioid-related side effects like PONV. These effects have been demonstrated across many realms of surgery, including otolaryngology, abdominal surgery, orthopedic surgery, spinal surgery, and gynecological surgery. While all of the exact mechanisms have not been entirely agreed upon, the drugs' primary effect is related to its NMDA receptor antagonism. An intravenous bolus given before incision followed by a continuous infusion is the most effective modality for postoperative pain control. Suppose the injection is provided over a protracted time course (48 h) for more invasive and routinely painful procedures. In that case, patients can have a decreased risk of developing persistent postoperative pain in the months that follow.

To the best of the authors' knowledge, no other study has ever examined the effect of local infiltration of ketamine at the site of incision on postoperative pain after hysterectomy. Therefore, this study was performed to determine the impact of a low dose ketamine infiltration in the wound on postoperative analgesia.

## Figures and Tables

**Table 1:** Comparison of pain intensity according to the visual analog scale at different intervals in the two study groups.

Time interval (h)	Case group (*n* = 46)	Control group (*n* = 46)	*P* value
1	6.52 ± 1.79	8.23 ± 1.62	0.001^*∗*^
2	6.02 ± 1.77	7.52 ± 2.06	0.001^*∗*^
4	5.61 ± 1.56	6.91 ± 1.44	0.001^*∗*^
6	5.74 ± 0.99	7.63 ± 1.94	0.001^*∗*^
12	4.93 ± 1.95	8.02 ± 1.51	0.04^*∗*^
24	4.59 ± 1.10	5.00 ± 1.03	0.06

Data are presented as the mean ± SD. ^*∗*^*P* value <0.05 is statistically significant.

**Table 2:** Comparison of doses of administered morphine at different intervals in the two study groups.

Time interval (h)	Case group (*n* = 46)	Control group (*n* = 46)	*P* value
1	1.84 ± 2.44	2.72 ± 2.52	0.96
2	1.74 ± 2.41	2.28 ± 2.52	0.29
4	1.74 ± 2.41	1.74 ± 2.41	0.90
6	1.63 ± 2.37	2.93 ± 2.49	0.01^*∗*^
12	3.04 ± 2.47	4.02 ± 2.01	0.04^*∗*^
24	2.07 ± 2.49	2.61 ± 2.53	0.30
Total	11.96 ± 3.57	15.98 ± 3.27	0.001^*∗*^

Data are presented as the mean ± SD. ^*∗*^*P* value <0.05 is statistically significant.

## Data Availability

The datasets used to support the findings of this study are available from the corresponding author on reasonable request.
